# Engineered Shape-Tunable Copper-Coordinated Nanoparticles for Macrophage Reprogramming

**DOI:** 10.1021/acs.nanolett.4c05999

**Published:** 2025-02-06

**Authors:** Han Gao, Ruoyu Cheng, Inês Cardoso, Maria Lobita, Idaira Pacheco-Fernández, Raquel Bártolo, Lígia R. Rodrigues, Jouni Hirvonen, Hélder A. Santos

**Affiliations:** † Department of Biomaterials and Biomedical Technology, The Personalized Medicine Research Institute (PRECISION), University Medical Center Groningen, University of Groningen, Ant. Deusinglaan 1, 9713 AV Groningen, The Netherlands; ‡ Drug Research Program, Division of Pharmaceutical Chemistry and Technology, Faculty of Pharmacy, University of Helsinki, FI-00014 Helsinki, Finland; § CEB - Centre of Biological Engineering, Universidade do Minho, Campus de Gualtar, 4710-057 Braga, Portugal

**Keywords:** Immune response, Nanoparticles, Shape-tunable, Copper coordination nanocomplexes, Macrophage reprogramming

## Abstract

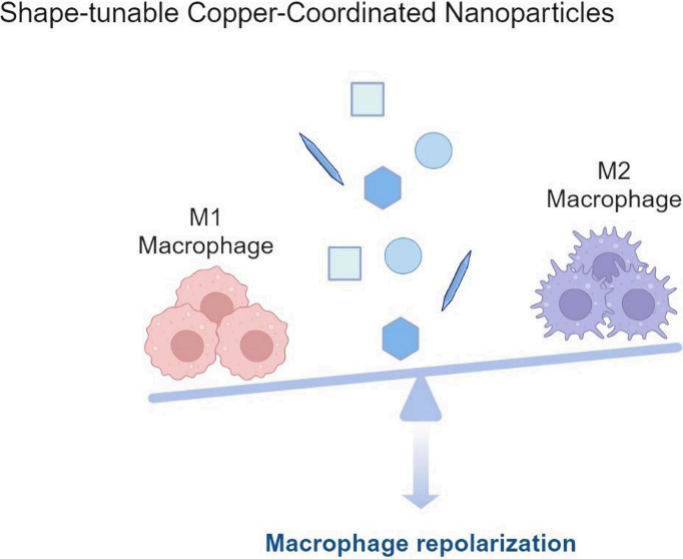

The immune system safeguards as primary defense by recognizing nanomaterials and maintaining homeostasis, gaining a deeper understanding of these interactions may change the treating paradigm of immunotherapy. Here, we adopted copper as the principal component of nanoparticles (NPs), given its features of coordination with different benezenecarboxylate ligands to form metal–organic frameworks and complexes with distinct morphologies. As a result, four types of shape-tunable copper-coordinated NPs (CuCNPs) are developed: cuboctahedron, needle, octahedron, and plate NPs. Biocompatibility of CuCNPs varies across different cell lines (RAW264.7, THP-1, HEK 293 and HeLa) in a shape-dependent manner, with needle-shaped CuCNPs showing pronounced cytotoxicity (IC50:104.3 μg mL^–1^ at 24 h). Among different shapes, a notable increase of 8.47% in the CD206^+^ subpopulations is observed in needle-shaped CuCNPs, followed by 77% enhancement at 48 h. Overall, this study underscores the shape-dependent immune-regulatory effects of CuCNPs and sheds light on the rational design of nanoscale metal complexes for potential immunotherapy.

The immune system plays a crucial role in the pathogenesis of various diseases, serving as the host defense mechanism against foreign invaders, such as pathogens (e.g., bacteria, viruses, fungi, and parasites), as well as abnormal cells (e.g., cancer cells).^1^ Of particular interest, modulating immune system holds great potential for tailored treatment of various conditions, especially in the scenario of inflammatory diseases.^1^ In this context, nanomaterials have been extensively investigated for their capacities to modulate immunological processes via harnessing specific physicochemical or biological properties.^2^ For example, cell membrane nanomaterial engineering enables the direct modulation of antigen-specific T cell populations, leveraging immune therapy for controlling tumor growth.^3^

Metal-coordination complexes (MCCs) are a group of materials that combine the functions of metal ions and organic molecules via coordination strategies.^4,5^ In recent decades, rapid growth in coordination chemistry has led to numerous advances in the field and the description of new structures, such as metal–organic frameworks (MOFs).^6^ Because of their synthetic versatility and tunable properties they have been found useful in a broad range of applications that encompass biosensing, catalysis and immunotherapeutics.^5,7,8^ Recently, our group has demonstrated the potential of a tannic acid–iron(III) complex in the development of formulations for immunotherapy and antifibrosis within atherosclerotic plaque.^9^ Likewise, copper-based MCCs have been extensively studied in the fields of modulating oxidative stress,^10^ regulating immune responses,^11^ inhibiting proteasomes,^12,13^ and facilitating bioimaging.^14,15^

Despite emerging investigations into the application of MCCs for achieving the desired therapeutic outcomes, the underlying understanding of how cells interact with nanostructures of well-defined shapes remains poorly understood. Within this framework, the preparation of MCCs with specific features can be finely tuned by selecting the proper organic linkers and the metal center or by adjusting the coordination geometries.^4^ As a result, differences in physicochemical properties may induce different biological outcomes, such as cytotoxicity and immune response.^16,17^ Recently, it has been demonstrated that the shape of nanoparticles (NPs) can significantly impact their performance toward T-cell immune responses modulation.^18^ As an example, a previous study reported that nanospheres were most potent at inducing IgG2a antibody responses whereas nanorods induced IgG1 antibody responses.^19^ Moreover, the shape can modulate the uptake of NPs, with triangles showing the most efficient cellular uptake in macrophages.^20^ The variations in cellular interactions, such as changes in receptor–ligand binding, can trigger a cascade of innate inflammatory responses.^21^ Overall, given the active involvement of engineered NPs in biological regulatory processes, we postulate the potential for modulating immune responses through the manipulation of particles’ morphology.

In this study, we investigated the effects of diverse NPs’ morphologies on the immunological response (Scheme 1). Copper-containing MCCs were prepared via coordinating various benzenecarboxylate ligands, yielding MOFs and metal complexes with four distinct morphologies: needle, cuboctahedron, octahedron, and plate. Comprehensive biocompatibility evaluations were conducted across multiple cell lines followed by cytotoxicity assessments. Utilizing an established immune cell model, we examined the immunomodulatory effects of these shape-tunable copper-coordinated NPs (CuCNPs), focusing particularly on macrophage repolarization and the immune response toward M1-type macrophages. This work provides an in-depth examination of the immune responses triggered by shape-tunable CuCNPs, offering insights into the rational design of metallic nanocomplexes for potential applications in immunotherapy.

**Scheme 1:**
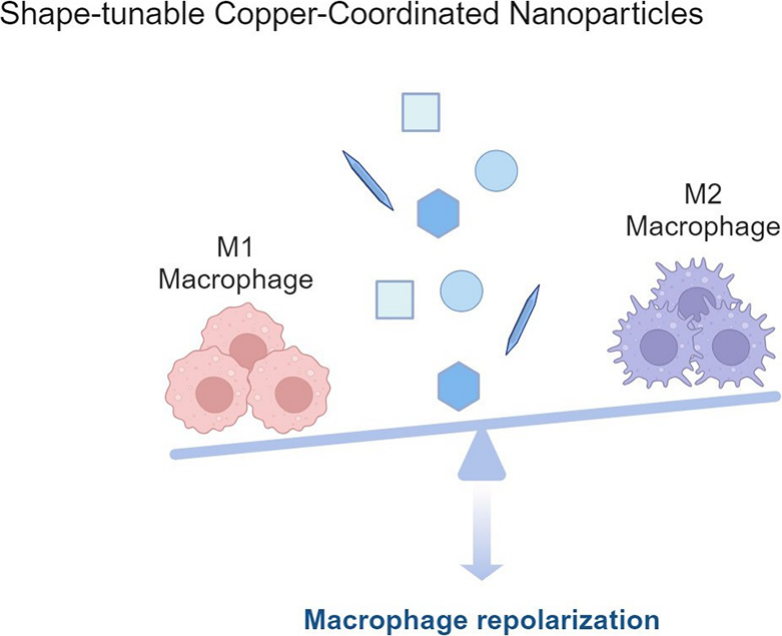
Schematic Illustration of Shape-Tunable Copper-Coordinated Nanoparticles (CuCNPs) on Modulating Immune Responses (Created with BioRender.com)

## Characterization of Shape-Tunable Copper-Coordinated NPs

**Figure 1:**
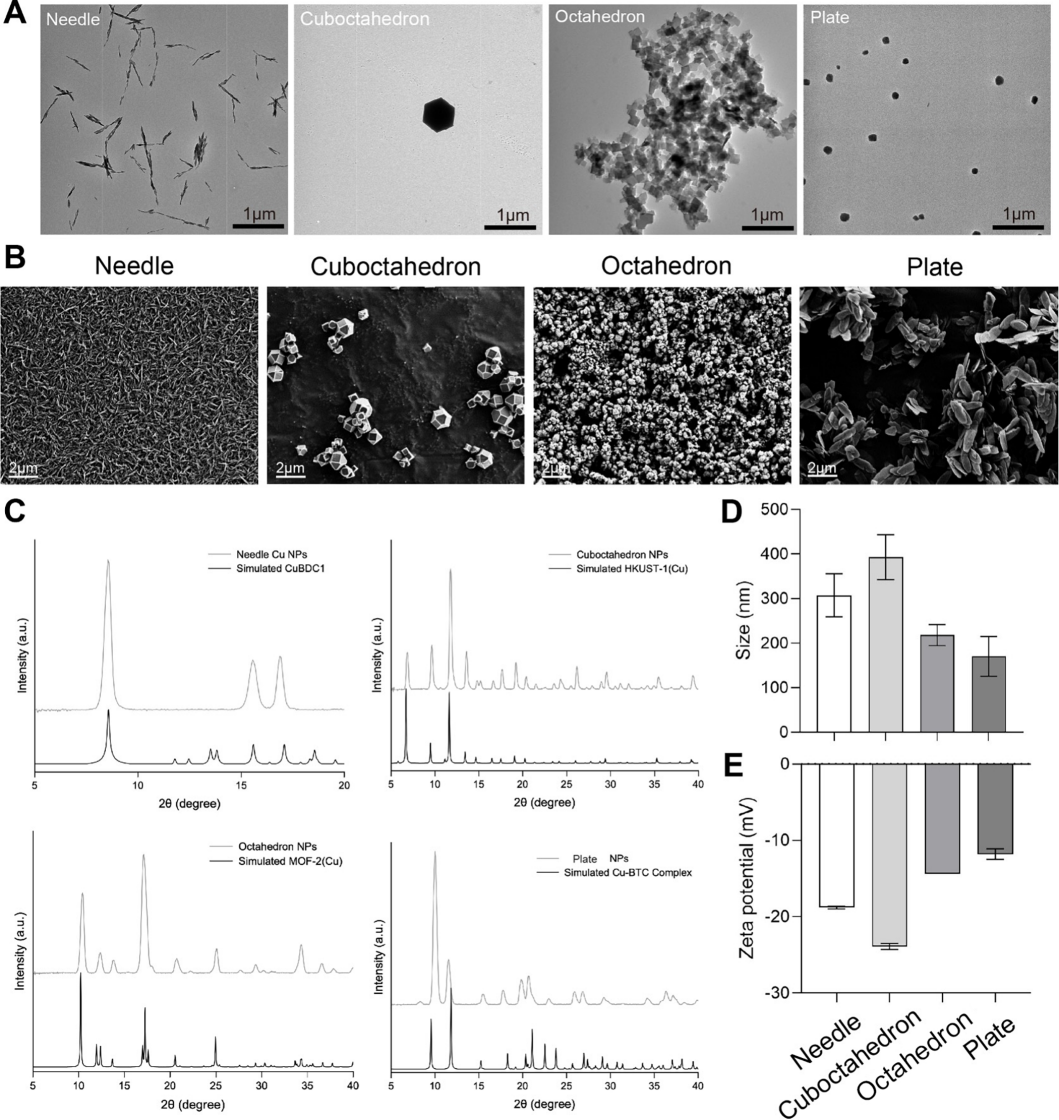
Preparation and characterization of shape-tunable copper-coordinated nanoparticles. (A) The morphology of four shape-tunable CuCNPs was observed via TEM imaging. Scale bar: 1 μm. (B) The surface morphology of shape-tunable CuCNPs was analyzed via SEM imaging. Scale bar: 2 μm. (C) PXRD characterization of shape-tunable CuCNPs for determining the crystalline phase. (D) Size (nm) and E. Zeta-potential were determined using DLS analysis. Data are shown as mean ± SD.

Among the wide variety of organic ligands that can be used to synthesize CuCNPs, carboxylate ligands are the most common because of their high stability, in contrast with azole or pyridine ligands. In particular, benzenecarboxylate with multiple carboxylic groups are more desirable to obtain structures with improved stability and rigidity due to the higher connectivity of metal centers and the reduced interpenetration.^22,23^ Thus, in this study, benzenecarboxylic acids with 2 to 4 carboxylic groups were used as ligands to prepare the CuCNPs. Diverse modified protocols from the literature were used to obtain NPs with different shapes to evaluate the effect of this feature in the immune response while keeping the same NPs composition as much as possible. The morphologies of as-designed CuCNPs were characterized using TEM analysis, indicating the successful fabrication of four nanoformulations with distinct shapes, including needle, cuboctahedron, octahedron and plate (Figure 1A). To gain more information about the surface morphology of the developed nanoparticles, SEM analysis was conducted. Figure 1B illustrates the homogeneous dispersion of the synthesized CuCNPs, each exhibiting discernible morphologies, aligning consistently with the observations captured via TEM. Lower magnification images are shown in Figure S1 to offer a broader perspective of the samples.

Once NPs with different shapes were obtained, their structural characterization was carried out. Considering the coordination nature of the NPs, PXRD characterization was carried out to determine their crystalline phase. For the octahedron and cuboctahedron CuCNPs, their PXRD patterns were compared with the simulated patterns of the MOFs consisting of the coordination of Cu with BDC and BTC ligands, respectively. Thus, as shown in Figure 1C, the octahedron NPs correspond to the MOF-2(Cu) (CCDC 687689),^24^ while the cuboctahedron NPs matches the pattern for the HKUST-1(Cu) MOF (CCDC 755080).^25^ In the case of the needle-shaped CuNPs composed of Cu and BDC ligands, the pattern did not align with the simulated pattern for conventional MOF-2(Cu) synthesized in *N*,*N*-dimethylformamide (CCDC 687690). This matched the patten reported for a layered Cu-BDC MOF (CuBDC1, obtained by modifying CCDC 898032) that crystallizes in the monoclinic space *C*2/*m* but without coordinated solvent molecules, which is consistent with the synthetic conditions used in the present study (Figure 1C).^26,27^ The formation of this MOF phase in contrast to the octahedron NPs of MOF-2(Cu) might be related to the presence of the 2-MIm modulator used during the synthesis of the needle-shape NPs, which deprotonates the ligand and accelerates the nucleation process but also acts as competing ligand to control the growth of the MOF in the stacked layers.^28^ For the plate CuCNPs, the PXRD pattern perfectly matches the simulated pattern for the Cu coordination complex with H_4_BTC as shown in Figure 1C (CCDC 652500).^29^

The physicochemical properties of CuCNPs were characterized by DLS, revealed a hydrodynamic diameter of 307.5 ± 48.4 nm for needle-shaped CuCNPs, 392.8 ± 50.3 nm for cuboctahedron-shaped CuCNPs, 218.1 ± 23.5 nm for octahedron-shaped CuCNPs and 170.7 ± 44.7 nm for plate-shaped CuCNPs (Figure 1D). Moreover, zeta potential analysis revealed that all copper-based NPs displayed negative surface charges, suggesting the feasibility of conducting parallel comparisons for subsequent biological evaluations (Figure 1E). NTA was conducted by maintaining consistent particle concentrations across different CuCNPs (Table S2).

Altogether, these results demonstrate the successful construction of four shape-tunable CuCNPs with appropriate physicochemical properties, which are feasible for subsequent investigations.

## Biocompatibility of CuCNPs

The interaction between nanomaterials and cellular systems is closely associated with the molecular structure, formulation, colloidal stability and mechanical properties of NPs.^30^ As a prerequisite, the evaluation of nanomaterials biocompatibility is crucial for determining the potential biological effects.^31^ Considering this, we proceeded to evaluate the cytotoxic effects of the synthesized CuCNPs across various cell lines, including RAW264.7, THP-1, HeLa, and HEK 293 cells. These cell lines were selected to represent a broad spectrum of biological contexts, including immune response, oncogenicity, and immortalized kidney function, thereby offering comprehensive insights into the potential impacts on diverse cellular systems.^32−34^

**Figure 2:**
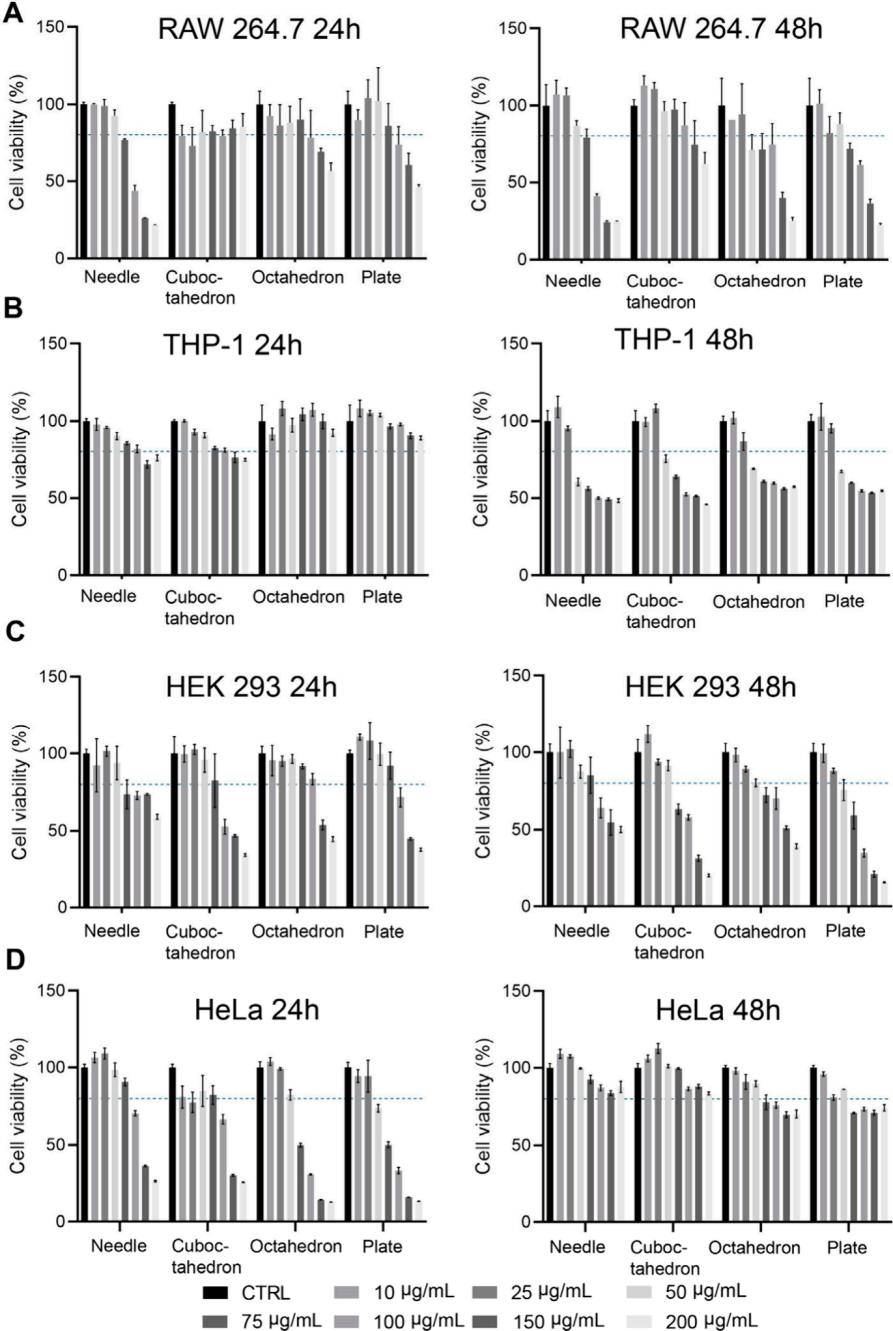
Cytotoxicity of shape-tunable CuCNPs on distinct cell lines. Four shape-varying CuCNPs were incubated with different types of cell lines, including (A) RAW264.7 cells, (B) THP-1 cells, (C) HEK 293 cells and (D) HeLa cells. The working concentrations ranged from 0 to 200 μg mL^–1^. Data are shown as mean ± SD. The results were analyzed with two-way ANOVA, followed by Tukey’s post-test within two groups.

In vitro biocompatibility was examined using the AlamerBlue assay to monitor the cell viability. Briefly, cells were exposed to varying concentrations of Cu-based nanoformulations, ranging from 10 to 200 μg mL^–1^, and after 24 and 48 h, the cytotoxicity was evaluated. As shown in Figure 2, CuCNPs with diverse shapes exhibit differential biocompatibility profiles toward cells. In RAW264.7 macrophages, needle-shaped CuCNPs showed statistically significant cytotoxicity when concentrations surpassed 75 μg mL^–1^, following a 24 h incubation with the cells. By extension of the incubation period to 48 h, all tested nanoformulations demonstrated dose-dependent cytotoxicity at concentrations exceeding 100 μg mL^–1^ (Figure 2A). In contrast, this phenomenon was not observed in THP-1 derived macrophages, wherein detectable toxicity was only evident after 48 h of incubation with shape-tunable CuCNPs (Figure 2B). This can be attributed to the divergent proteomic patterns and sensitivity within different macrophage phenotypes.^35,36^

Moreover, in HEK 293 human embryonic kidney cells, the assessed CuCNPs were cytocompatible up to 100 μg mL^–1^, with a time-dependent toxicity profile when the incubation time was increased to 48 h (Figure 2C). This is likely attributed to the exposure time of NPs,^37^ over the incubation period; augmented accumulation of NPs within cells may trigger cellular stress, consequently amplifying cytotoxicity levels.^38^ In contrast, in the case of HeLa cells, we noted a divergent pattern, wherein cells exposed to shape-tunable CuCNPs demonstrated an initial decline in viability at 24 h, followed by recovery at 48 h (Figure 2D). This can be ascribed to the compensatory proliferation of tumor cells. Previous studies have shown that cells induced to undergo apoptosis can activate nearby surviving cells, potentially leading to tumor regrowth or recurrence.^39^

Altogether, these results indicate shape-dependent cytotoxic effects of CuCNPs. The IC50 values of shape-dependent CuCNPs after 24 h of incubation across different cell lines were presented in Table S3. In the following studies, the NP concentration of 75 μg mL^–1^ was used in the relevant cell line for further investigations on the immune response.

## Immune-Regulatory Capability of CuCNPs

**Figure 3:**
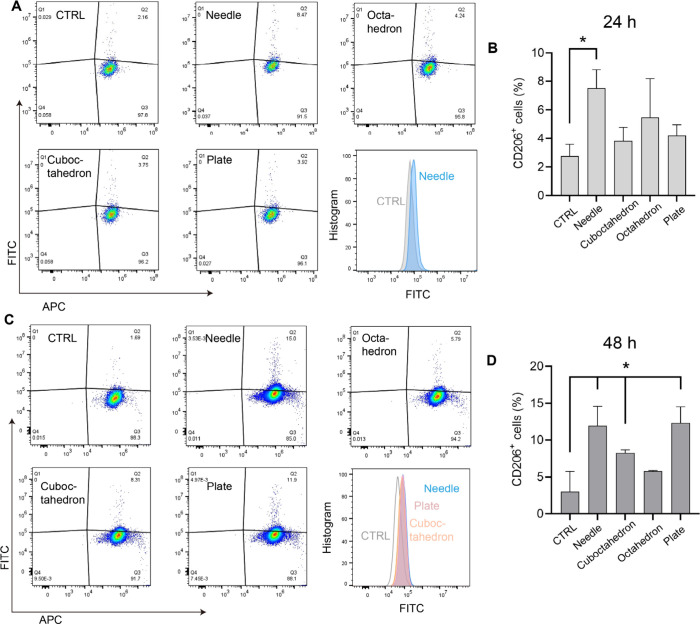
Shape-tunable CuCNPs regulate macrophage repolarization. Four shape-varying CuCNPs were incubated with RAW264.7 macrophages at the concentration of 75 μg mL^–1^ for 24 h (A, B) and 48 h (C, D). Flow cytometry analysis was performed to analyze the repolarization effects.

Given the potential shape-dependent immunomodulatory effects of nanomaterials, we next investigated whether the prepared CuCNPs would trigger the maturation transition of macrophages. RAW264.7, the most used murine macrophage, was chosen as model cell line to explore the hypothetical immune response.^40^ In the scenario of inflammatory conditions, macrophages with inflamed status represent the target cell type for personalized treatments.^41^ Thus, we sought to evaluate the phenotype-tuning capabilities of CuCNPs by incubating them with M1-like macrophages. As shown in Figure 3, the expression level of the co-stimulatory marker CD206 was quantified through flow cytometry analysis, which is the representative of the activation state M2-type macrophages.^42^Figure 3A shows the expression profiles of CD86 and CD206 on RAW264.7 macrophages preincubated with 75 μg mL^–1^ shape-varying CuCNPs, and the gating strategy is shown in Figure S2. Compared to other experimental groups, a statistically significant augmentation of CD206 expression was evident in M1-like macrophages treated with needle-shaped CuCNPs, as quantified by a notable increase of 8.47% in the CD206^+^ subpopulations, indicating its potential anti-inflammatory effects toward innate immune response. A similar trend was observed in cells treated with cuboctahedron-/plate-shaped CuCNPs, whereas negligible significance was detected at this time point (Figure 3B).

To gain insight into the immune-regulatory effects of the developed shape-tunable CuCNPs, we further increased the incubation period from 24 to 48 h. As depicted in Figure 3C, with prolonged incubation time, more pronounced immune-regulatory effects were observed in the tested CuCNPs. In particular, the enhancement of CD206 by 77% in needle-shaped CuCNPs was quantified compared to that with 24 h treatment. Concurrently, the anti-inflammatory effect was notably potentiated in plate-shaped CuCNPs, as indicated by an 11.9% increase in CD206 expression. A slight increase of CD206 to 8.31% was noted in cells incubated with cuboctahedron-shaped CuCNPs, which was comparable to that in the control group. In contrast, no statistically significant increase in M2 macrophages was observed in the group treated with octahedron-shaped CuCNPs within the time frame of our study (Figure 3D).

Overall, these results show the potential immunomodulatory effects of shape-tunable CuCNPs, with a time-dependent augmentation of the M2-phenotype cell populations.

## Determination of Gene Profile in Murine Macrophages

An initial proof of the macrophage repolarization effects of shape-tunable CuCNPs was presented by labeling cells with antibodies against specific surface markers (Figure 3). Nevertheless, systematic evaluation of developed nanoformulations in vitro is a crucial prerequisite for further clinical translation.^9^ Thus, to further elucidate the modulatory immune response induced by shape-tunable CuCNPs, we next investigated the gene expression profiles of key markers within macrophages. In this context, genes associated with M2 polarization, including CD206 and Arginase-1 (Arg-1), were detected. RAW264.7 macrophages were incubated with different shape-based CuCNPs at 75 μg mL^–1^, followed by determination of phenotype-specific biomarkers via quantitative reverse transcription polymerase chain reaction (RT-qPCR), the housekeeping gene GAPDH was used as internal control. The concentration and purity of extracted RNA are shown in Table S4.

**Figure 4:**
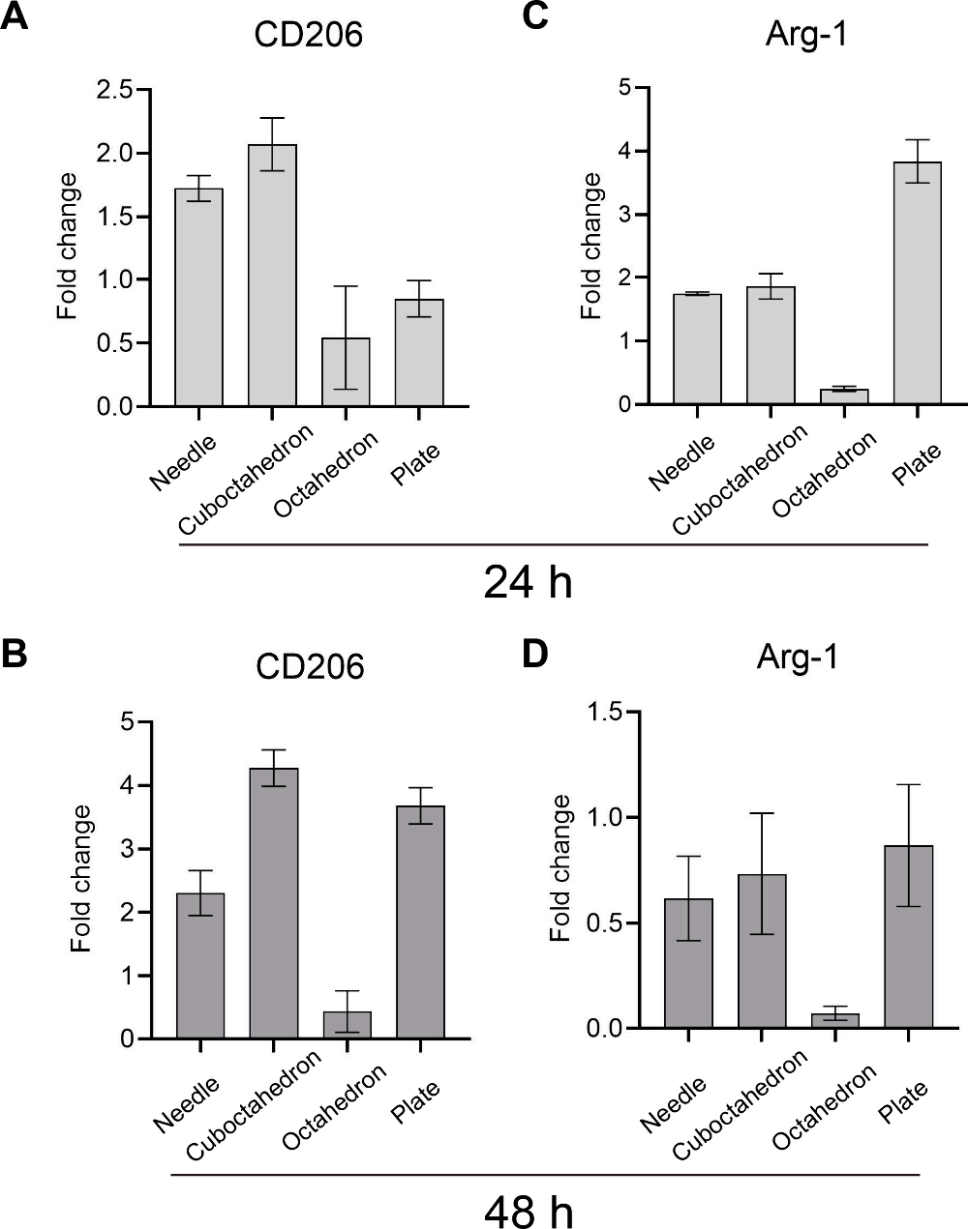
Gene expression levels of biomarkers for M2-phenotype macrophages. (A, C) The gene expression levels of CD206 and Arg-1 after treatment with shape-switching CuCNPs for 24 h. (B, D) The gene expression levels of CD206 and Arg-1 after treated with shape-switching CuCNPs for 48 h. Data are shown as mean ± SD *, *p* < 0.05. The results were analyzed with two-way ANOVA, followed by Tukey’s post-test within two groups.

As shown in Figure 4A, significantly enhanced expression of CD206 was observed within both the needle-shaped and plate-shaped CuCNPs groups. Moreover, a time-dependent trend was found, indicated by the fold change increasing from 1.7 to 2.3 in needle-shaped CuCNPs, and from 0.8 to 3.8 in plate-shaped CuCNPs (Figure 4B). A similar pattern was also observed in cuboctahedron-shaped CuCNPs, as evidenced by a fold change of 2.1 at 24 h and 4.3 at 48 h (Figure 4B). This can be attributed to the high sensitivity of the PCR reaction.^43^ Contrarily, despite the augmented expression of Arg-1 at 24 h incubation, we did not notice a time-dependent effect within the three groups after 48 h of incubation (Figure 4C,D). The proposed mechanism for this phenomenon is attributed to the metabolic signature and subphenotype within M2-like macrophages, arginase competes with inducible nitric oxide synthase (iNOS) for arginine, which in turn block NO production in the cells.^44^

Altogether, these results are consistent with the aforementioned flow cytometry analysis (Figure 3), showing the capacity of shape-tunable CuCNPs to harness immune responses.

## Expression Profiles of Inflammatory Cytokines

**Figure 5:**
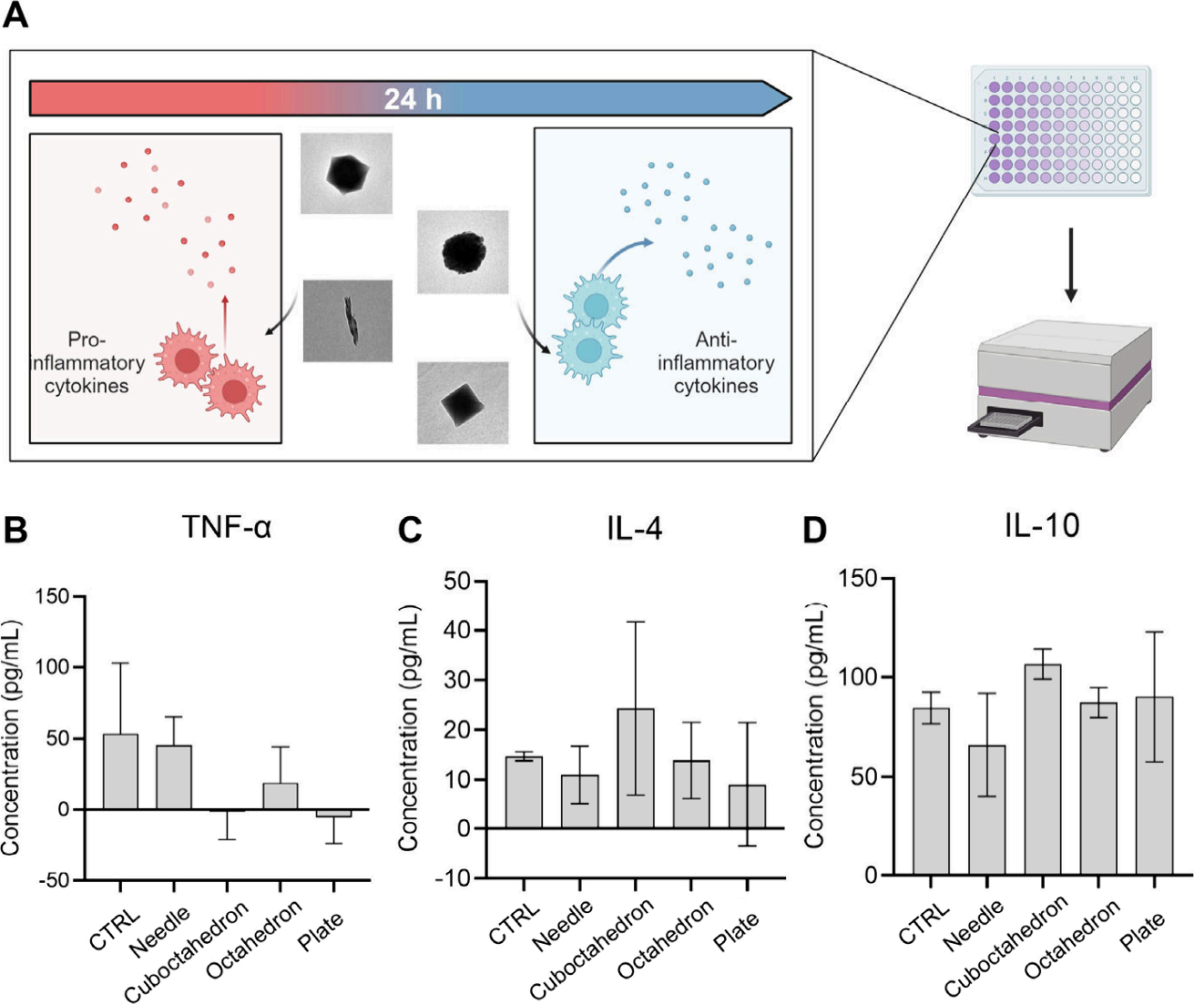
Comparison of cytokines expression profiles. (A) Experimental illustration of distinct effects of shape-tunable CuCNPs on RAW264.7 macrophages. Cytokine expression levels, including (B) TNF-α, (C) IL-4 and (D) IL-10 were determined by ELISA assay. Data are shown as mean ± SD. The results were analyzed with two-way ANOVA, followed by Tukey’s post-test within two groups.

The production of inflammatory cytokines occurs concomitantly with the initiation of macrophage polarization.^45^ The ability of shape-tunable CuCNPs on modulating the secretion of pro/anti-inflammatory cytokines, including TNF-α, IL-4 and IL-10, was then explored. Consistent with the NPs treatment conditions, cells were incubated with shape-varying CuCNPs at 75 μg mL^–1^ for 24 h, followed by the quantification of distinct cytokines using Enzyme-Linked Immunosorbent Assay (ELISA) (Figure 5A). As depicted in Figure 5B, a suppression of TNF-α was evident in both cuboctahedron- and plate-shaped CuCNPs groups, which was consistent with the results observed from qPCR analysis. CuCNPs with cuboctahedron/plate shapes triggered the expression of CD206 and Arg-1, the hallmark cytokines correlated with wound healing and tissue regeneration; as a result, the suppressive activity on TNF-α was more pronounced in these two groups and suggested a negative regulatory effect of TNF-α on Arg-1 expression in macrophages. In contrast, a marginal decrease of TNF-α was observed in needle- and octahedron-shaped CuCNPs.

Moreover, the anti-inflammatory cytokines IL-4 and IL-10 were assessed across different groups, as shown in Figure 5C,D, whereas only a slight increase of IL-4 expression was observed in cuboctahedron-shaped CuCNPs, which can be attributed to the elevated percentage of M2-type macrophage.

Altogether, these results further demonstrate the capability of shape-tunable CuCNPs to potentially regulate innate immunity.

Nanomaterials are revolutionizing the transformative paradigm in the landscape of biomedical applications, opening up new avenues for personalized medicine.^46^ By harnessing the distinctive characteristics of NPs, it holds the promise for achieving controlled sustained drug releases and targeted organ tropism.^46,47^ Nevertheless, with extensive research navigated on the discovery of NPs as drug delivery system, the intricate interplay between nanoscale shape biology and the immune system remains overlooked.^48^ Despite extensive research, few nanomedicines have been clinically translated, highlighting the need to understand NPs’ shape-dependent effects on innate immunity.^46,49^

In this study, leveraging coordination-driven complexing for the design of metal–organic NPs,^50^ we synthesized four shape-varied NPs originating from the preorganized geometries of benzenecarboxylate ligands and copper ions. The obtained NPs showed distinct morphologies (including needle, cuboctahedron, octahedron, and plate) with negative charge. Yet, despite carboxylates being prevalent in constructing porous hybrid materials, the resultant metallic complexes may vary depending on the geometry of the carboxylic groups. Upon this, the phase identification of as-synthesized biomaterials was determined by the PXRD technique, which is well-established for determining the structures of crystalline materials. With this, two branches of coordination polymers were identified: MOFs and metal–organic complexes. The former group is composed of MOF HKUST-1(Cu) (cuboctahedron shape), MOF-2(Cu) (octahedron shape), and a layered Cu-BDC MOF (needle shape), while the latter encompasses a copper complex formed by 1,2,4,5-benzenetetracarboxylate ligands placed in alternate faces. Of note, the layered structure of the Cu-BDC MOF phase was observed from the PXRD patterns of the needle-shaped CuCNPs, which was previously reported only when the MOF-2(Cu) was synthesized on modified substrates by a liquid-phase epitaxy approach or by electrochemical methods in *N*,*N*-dimethylformamide, but not by the conventional solvothermal methods.^26,27^ This may be attributed to the synthesis conditions used here, in which 2-MIm ligand was used for modulation and water and ethanol were used as synthesis solvents.^51^

From the cytotoxicity results, it is evident that CuCNPs of various shapes exert differential effects on cell viability, of which the needle-shaped NPs exhibited more toxicity than those within the comparison pool. These results are consistent with previous reports, indicating the pronounced toxicity derived from needle-shaped NPs, potentially attributed to their propensity for endocytic mechanisms and adhesion to the cellular surface.^52−54^

Macrophages serve as host defenders to maintain homeostasis and mounting immune responses against invading pathogens.^55^ Upon stimulation, macrophages exhibit remarkable plasticity, swiftly altering their metabolic and functional states in response to external stimuli.^55^ In this context, macrophages in response to varied environmental conditions are polarized into different subtypes, including M1- and M2-macrophages.^56^ The dysregulation of M1/M2 macrophage subpopulations plays a pivotal role in the initiation, perpetuation, and resolution of chronic inflammation.^55,57^ We observed a clear shape-dependent immune response; for needles, significantly higher CD206 subpopulations (8.5%) were already observed at 24 h, which was further increased to 15.0% by prolonging the incubation time. Other studies have also indicated that needle-shaped nanoparticles can effectively interact with the immune cells and elicits alterations in the immunological reaction.^58,59^ Moreover, the time-dependent immune-regulatory effects were observed from the cuboctahedron and plate-shaped CuCNPs, in which nanospheres showed higher M2-like macrophage subpopulations than that with nano cuboctahedrons.

Cytokines released from innate immune cells are critical messengers that initiate and constrain inflammatory responses to pathogens and injury.^60^ Pro-inflammatory cytokines, exemplified by tumor necrosis factor-alpha (TNF-α) and interleukin-12 (IL-12), possess the capability to elicit innate immune responses by orchestrating the recruitment of additional immune cells and facilitating the activation and maturation of adaptive immune cells,^60,61^ whereas anti-inflammatory cytokines, such as interleukin-10 (IL-10) and transforming growth factor-β (TGF-β), elicit immunosuppressive effects by attenuating the production of pro-inflammatory cytokines and fostering the resolution of inflammation. NPs with different morphologies modulated the expression profile of cytokines, such as TNF-α, IL-4 and IL-10. Manipulating the shape of the developed CuCNPs resulted in a statistically significant suppression of TNF-α cytokine secretion, which was attributed to the shape-specific effects.

In conclusion, this study highlights the pivotal role of nanoparticles’ morphology in modulating immune responses. Our findings reveal that plate copper-coordinated NPs elicit significantly stronger immune-regulatory effects than other shapes, suggesting a unique shape-dependent influence on immune function. Additionally, our study underscores the broader significance of NP design, particularly in tailoring nanostructures to fine-tune immune responses. This work provides valuable guidance for the future development of hybrid porous nanomaterials with targeted immunomodulatory properties, advancing their potential applications in immunotherapy and other biomedical fields.

## Supporting Information Available

Experimental details, SEM images, gate strategy for flow cytometry analysis, PCR primer sequences, Concentration of NPs, IC50 values of NPs across different cell lines, and total RNA concentration (PDF)
