# Supplementary material for: Engineered Shape-Tunable Copper-Coordinated Nanoparticles for Macrophage Reprogramming
Source: Nano Lett. 2025 Feb 6;25(7):2831–40. doi: 10.1021/acs.nanolett.4c05999 (PMC11849021; doi:10.1021/acs.nanolett.4c05999)
Supplement: Supplementary file 1 — nl4c05999_si_001.pdf [file nl4c05999_si_001.pdf]

Supporting Information for

Engineered shape-tunable copper-coordinated nanoparticles for macrophage reprogramming

Han Gao[1,2,], Ruoyu Cheng[1,2], Inês Cardoso[1,3], Maria Lobita[1], Idaira Pacheco-Fernández[1], Raquel Bártolo[1], Lígia R. Rodrigues[3], Jouni Hirvonen[2], Hélder A. Santos[1,2,*]*

1. Department of Biomaterials and Biomedical Technology, The Personalized Medicine Research Institute (PRECISION), University Medical Center Groningen, University of Groningen, Ant. Deusinglaan 1, 9713 AV Groningen, The Netherlands.
2. Drug Research Program, Division of Pharmaceutical Chemistry and Technology, Faculty of Pharmacy, University of Helsinki, FI-00014 Helsinki, Finland.
3. CEB - Centre of Biological Engineering, Universidade do Minho, Campus de Gualtar, 4710-057 Braga, Portugal.

Experimental section

Experimental reagents

Organic ligands including benzene-1,4-dicarboxylic acid (H_2BDC), 1,3,5-benzenetricarboxylic acid (H_3BTC) and benzene-1,2,4,5-tetracarboxylic acid (H_4BTC), 2-methylimidazole (2-MIm), copper (II) nitrate trihydrate [$Cu(NO_3)_2 \cdot 3H_2O$] were purchased from Sigma Aldrich (USA). All the reagents and solvents for the nanoparticle's synthesis are of ACS grade and supplied by Sigma Aldrich (USA). Ultrapure water was obtained from a purification system from Sartorius. AlamarBlue™ Cell Viability Reagent was obtained from Thermofisher, USA. The iScript™ cDNA Synthesis Kit and iQ SYBR Green Supermix were purchased from Bio-Rad, USA. Sequences of gene primers for PCR analysis were provided in **Table S1**. The ELISA kit for quantification of different cytokines secretion was obtained from PeproTech, USA.

Preparation of shape-tunable copper-coordinated nanoparticles (CuCNPs)

Needle-shaped CuCNPs: The synthesis followed a modified protocol described for the modulated synthesis of MOFs.[1] The metal solution was prepared by dissolving 10 mg (0.04 mmol) of $Cu(NO_3)_2 \cdot 3H_2O$ (10 mg) in 1 mL of ultrapure water (10 mg mL^{-1}). 40 mg (0.49 mmol) of 2-MIm were dissolved in 1 mL of ultrapure water (40 mg mL^{-1}) to prepare the modulator solution. For the ligand solution, 13 mg (0.08 mol) of H_2BDC were dissolved in a total volume of 4 mL of ultrapure water with the aid of 2-MIm to deprotonate the ligand (molar ratio 1:3.2). For NPs synthesis, 4 mL of absolute ethanol, 4 mL of ultrapure water, 945 µL of the metal solution and 1 mL of the ligand solution, were mixed, maintaining a metal-ligand molar ratio of 1:2. The reaction mixture was vigorously stirred (600 rpm) for 8 h at room temperature.

Octahedron-shaped CuCNPs: The synthesis was carried out following a previously reported method with slight modifications to reduce the particle size.[2] $Cu(NO_3)_2 \cdot 3H_2O$ (50 mg, 0.21 mmol) was dissolved in 5 mL of dimethylformamide (DMF) and 16 mg of H_2BDC (0.1 mmol) were dissolved in 4 mL of DMF. Then, 1 mL of the ligand solution, 604 µL of the metal solution and 3 mL of DMF were mixed to achieve a molar ratio of 1:1. The reaction mixture was vigorously stirred overnight at 80 °C using an oil bath. The resulting blue powder was isolated by centrifugation (Eppendorf Centrifuge 5430 R) and subjected to three washes with DMF (3 × 3 mL). Centrifugation was conducted at 7800 rpm and 4 °C for 20 min.

Cuboctahedron-shaped CuCNPs: To produce NPs with cuboctahedron shape, the following protocol was adapted from a previous study.[3] Briefly, Cu $(NO_3)_2.3H_2O$ (2.1 mg, 0.009 mmol) and the modulator octanoic acid (118.88 mg, 0.825 mmol) were dissolved in 10 mL of butanol. The mixed solution was heated with a heat gun until a transparent solution was obtained. H_3BTC (1 mg, 0.004 mmol) was added, and the mixture was heated by microwave irradiation at 413 K for 60 min. The resulting blue powder was isolated by centrifugation and washed with ethanol (3 × 3 mL). Centrifugation was performed at 7800 rpm, at 4 °C for 20 min.

Plate-shaped CuCNPs: A previous protocol with slights modifications was used.[4] A $Cu(NO_3)_2 \cdot 3H_2O$ solution at 0.04 M in DMF was prepared, while 16 mg of H_4BTC were dissolved in 4 mL of DMF to prepare the ligand solution. The reaction mixture consisted of 3 mL of DMF, 1 mL of the H_4BTC solution and 773 µL of the metal solution to keep a molar ratio of 1:2. The mixture was stirred overnight at 80 °C using an oil bath. The resulting product was centrifuged and washed with DMF (3 × 3 mL). Centrifugation was carried out at 7800 rpm and 4 °C for 20 min.

Nanoparticles characterization

The structural characterization of the NPs was carried out using a Bruker D8 Advance X-Ray diffractometer equipped with Cu-Kα radiation (1.5418 Å). Measurements were conducted over the range of 2º to 50º in increment steps of 0.020º and using a Si low background sample holder. The morphology of the NPs was characterized by using transmission electron microscopy (TEM) and scanning electron microscopy (SEM) analysis. The carbon-coated copper TEM grid (Ted Pella) was used for the preparation of nanoparticles and the TEM analysis was conducted by the FEI Tecnai G2 F20 X-TWIN Transmission Electron Microscope (USA). The hydrodynamic diameter, zeta potential and concentration of each shaped CuCNPs were determined via Zetasizer Nano ZS (Malvern) and nanoparticle tracking analysis (NTA).

Cell culture

Cell lines were cultured under specific conditions: RAW 264.7 (murine macrophages) in Dulbecco's Modified Eagle's Medium (DMEM) GlutaMAX™ supplemented with 10% Fetal Bovine Serum (FBS) and 1% Penicillin-Streptomycin (PS), HeLa cells were cultured in DMEM GlutaMAX™ supplemented with 10% FBS and 1% PS, HEK 293 cells were maintained in DMEM GlutaMAX™ containing 10% FBS and 1% PS. All cell lines were obtained from the American Type Culture Collection (ATCC) and authenticated prior to use. Cells were maintained in a 37°C incubator with 5% CO_2, sub-cultured every 2-3 days or upon reaching confluence. Sub-culturing involved detachment with a cell scraper or using trypsin-ethylenediaminetetraacetic acid (EDTA) and transfer into fresh culture flasks at specified ratios.

Cell viability test

Cell viability of RAW 264.7, HeLa, and HEK 293 cell lines were assessed after 24 h and 48 h of exposure to varying concentrations of CuCNPs using the AlamarBlue assay. Briefly, cells were seeded in 96-well plates at a density of 5×10^3 cells/well and allowed to reach 80% confluence before exposure to CuCNPs. Different concentrations of CuCNPs (ranging from 10 µg mL^{-1} to 200 µg mL^{-1}) were applied for 24 h and 48 h incubation periods at 37°C, followed by fluorescence determination using a plate reader (BioTek Synergy H1, Agilent). The corresponding cell viability

was calculated using the following equation:

$$\%Cell\ viability = \frac{Fluorescence_{sample}}{Fluorescence_{control}} * 100$$

Flow cytometry analysis

The polarization effects on macrophages treated with shape-tunable CuCNPs were evaluated via flow cytometry analysis. RAW 264.7 cells were seeded at a density of 2×10^5 cells/mL in 6-well plates and treated with four distinct types of CuCNPs at a concentration of 75 μg mL^{-1} for 24 h and 48 h. Subsequently, cells were harvested and subjected to wash with cold PBS. Afterwards, cells were blocked and incubated for 20 minutes at 4°C in a light-protected environment with APC-conjugated CD86 and FITC-conjugated CD206 antibodies. After two washes with staining buffer, cell polarity was assessed via flow cytometry. Flow cytometric data were acquired using a FAC Scan platform (Agilent NovoCyte Quanteon Flow Cytometer) and analyzed utilizing FlowJo (version 10) software. The purified antibodies used in the flow cytometry includes: FITC anti-mouse CD206 (MMR) Antibody (Biolegend, USA), APC anti-mouse CD86 Antibody (Biolegend, USA).

RNA extraction and Real-Time Polymerase Chain Reaction (qRT-PCR) analysis

Total RNA was extracted from RAW 264.7 cells treated with various groups of shape-tunable CuCNPs by using the InviTrap® Spin Universal RNA Mini Kit. RNA concentration and purity were assessed using a NanoDrop 1000 Spectrophotometer. RNA samples with OD260/OD280 ratios between 1.8 and 2.2 were utilized for cDNA synthesis with reverse transcriptase using the iScriptTM Bio-Rad cDNA Synthesis Kit. The resulting cDNA was diluted 1:20 with DNase-free water and then used in qRT-PCR reactions with iQ™ SYBR® Green Supermix. Reactions were set up using a pipetting robot and carried out in duplicate in a CFX384TM Real-Time system. Relative gene expression values were calculated using the ΔΔCt method, with data presented as $2^{-\Delta\Delta Ct}$.

Enzyme-linked Immunosorbent Assay (ELISA)

The ELISA assay is achieved by measuring the activity of the reporter enzyme after incubation with a substrate that produces the measurable products. Briefly, RAW 264.7 macrophages were treated with four different shapes of CuCNPs at 75 μg mL^{-1} for 24 h. After incubation, the culture media were collected for subsequent analysis of cytokine release. Pro-inflammatory cytokines (TNF-α and IL-12) and anti-inflammatory cytokines (IL-4 and IL-10) were measured using an ELISA kit (PeproTech TMB ELISA Buffer Kit) according to the manufacturer's instructions. Optical density (OD) at 450 nm was determined by using a plate reader (BioTek Synergy H1,

Agilent).

Statistical analysis

Results were analyzed using GraphPad Prism (version 9.4.0, GraphPad Software) and presented as means ± standard deviations (SD). Statistical differences among different groups were assessed using either Student's t-test or one-way analysis of variance (ANOVA) followed by Dunnett’s post hoc test. A p-value of ≤ 0.05 was considered statistically significant. Significant differences within groups were denoted in the graphs by asterisks (*), where *$p<0.05$, **$p<0.01$, ***$p<0.001$, and ****$p<0.0001$.

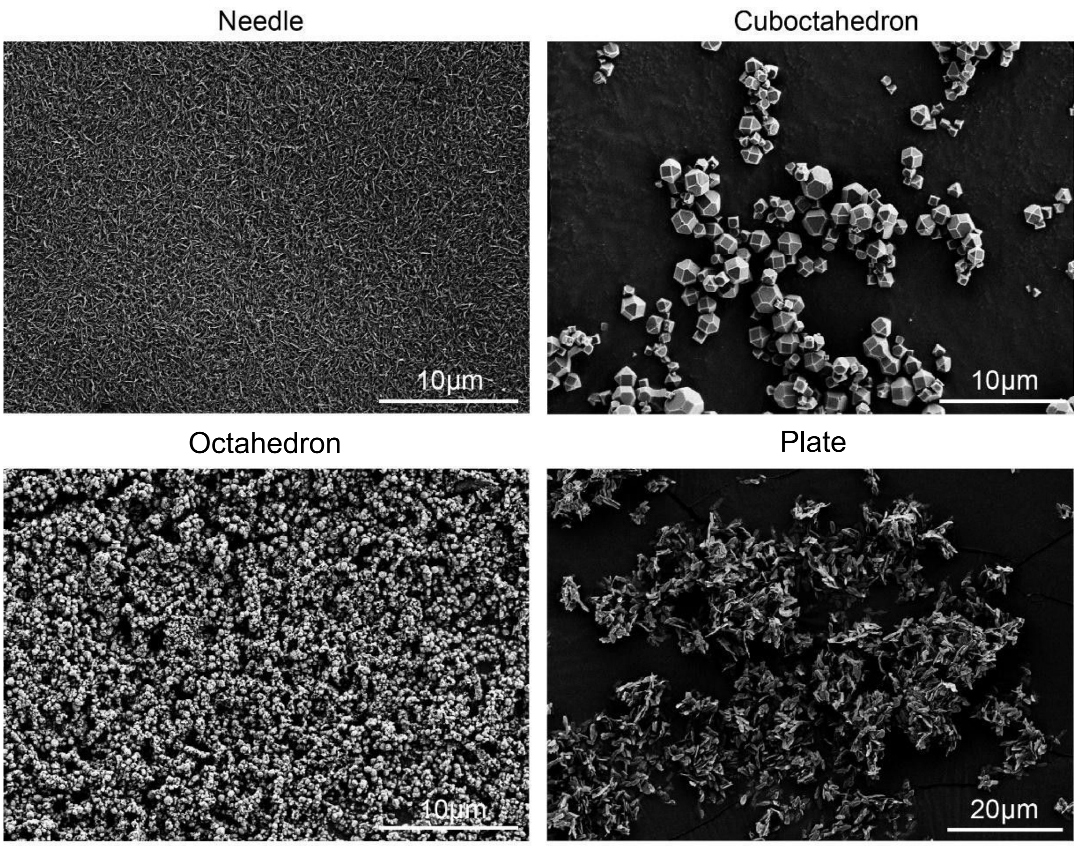

Fig. S1. SEM images with lower magnification, including needle/cuboctahedron/octahedron/plate-shaped CuCNPs.

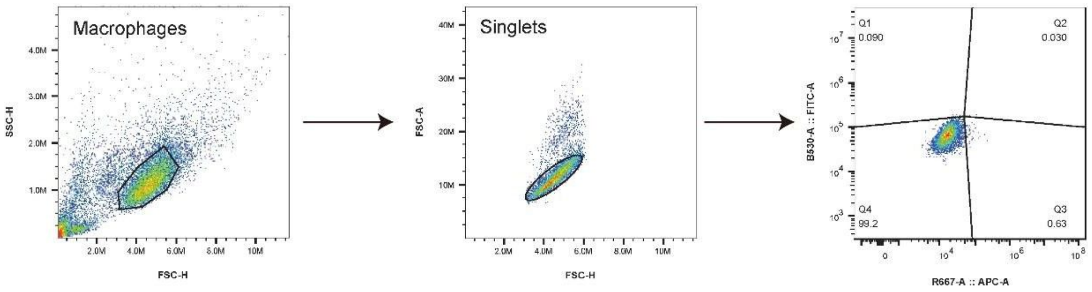

Fig. S2. Gating strategy for flow cytometry analysis.

Table S1. Sequences of primers for PCR analysis.

Primer	Forward 5'-3'	Reverse 5'-3'
CD206	GTGGTCCTCCTGATTGTGATAG	CACTTGTTCCTGGACTCAGATTA
Arg-1	TGGTGTGGTGGCAGA GGTCCA	ACTGCCAGACTGTGGTCTCCACC
GAPDH	GCATCCTGGGCTACACTGAG	TGGTCCTCAGTGTAGCCCAAG

Table S2. Concentrations of shape-tunable CuCNPs in nanoparticles tracking analysis

Groups	Concentration (particles/mL)
Needle-shaped CuCNPs	2.3×10^{11}
Cuboctahedron-shaped CuCNPs	1.7×10^{11}
Octahedron-shaped CuCNPs	2.1×10^{11}
Plate-shaped CuCNPs	0.6×10^{11}

Table S3. IC50 values of shape-dependent CuCNPs across various cell lines following 24-hour incubation.

	Needle	Cuboctahedron	Octahedron	Plate
RAW 264.7	104.3 μg/mL	—	—	181.4 μg/mL
THP-1	—	—	—	—
HEK 293	—	135.3 μg/mL	174.3 μg/mL	150.9 μg/mL
HeLa	134.0 μg/mL	122.3 μg/mL	78.30 μg/mL	75.99 μg/mL

Table S4. Total RNA concentration and purity assessed by NanoDrop Spectrophotometer.

Groups	**24 h**		**48 h**	
	Concentration (μg/ul)	$A_{260/280}$	Concentration (μg/ul)	$A_{260/280}$
**Control-1**	313.59	2.13	944.57	2.14
**Control-2**	204.37	2.15	1294.81	2.11
**Control-3**	382.98	2.11	1284.69	2.13
**Needle-1**	258.72	2.12	358.79	2.05
**Needle-2**	309.83	2.13	316.93	2.07
**Needle-3**	223.06	2.14	734.09	2.08
**Octahedron-1**	278.91	2.14	917.06	2.12
**Octahedron-2**	337.56	2.13	1372.81	2.11
**Octahedron-3**	300.18	2.12	426.23	2.07
**Plate-1**	375.49	2.13	1170.90	2.09
**Plate-2**	413.08	2.12	1105.22	2.11
**Plate-3**	254.89	2.15	928.68	2.12
**Cuboctahedron-1**	292.03	2.11	856.36	2.13
**Cuboctahedron-2**	330.65	2.12	1067.02	2.13
**Cuboctahedron-3**	269.57	2.15	1032.28	2.11

References

(1) Guo, C.; Zhang, Y.; Zhang, L.; Guo, Y.; Akram, N.; Wang, J. 2-Methylimidazole-Assisted Synthesis of Nanosized Cu3(BTC)2 for Controlling the Selectivity of the Catalytic Oxidation of Styrene. *ACS Appl Nano Mater.* **2018**, *1* (9), 5289-5296. DOI: 10.1021/acsanm.8b01283.
(2) Carson, C. G.; Hardcastle, K.; Schwartz, J.; Liu, X.; Hoffmann, C.; Gerhardt, R. A.; Tannenbaum, R. Synthesis and Structure Characterization of Copper Terephthalate Metal–Organic Frameworks. *Eur J Inorg Chem.* **2009**, *2009* (16), 2338-2343. DOI: https://doi.org/10.1002/ejic.200801224.
(3) Umemura, A.; Diring, S.; Furukawa, S.; Uehara, H.; Tsuruoka, T.; Kitagawa, S. Morphology Design of Porous Coordination Polymer Crystals by Coordination Modulation. *J Am Chem Soc.* **2011**, *133* (39), 15506-15513. DOI: 10.1021/ja204233q.
(4) Luo, J. H.; Huang, C. C.; Huang, X. H.; Chen, X. J. Two pseudo-polymorphic copper-benzene-1,2,4,5-tetracarboxylate complexes. *Acta Crystallogr C.* **2007**, *63* (Pt 6), m273-276. DOI: 10.1107/s0108270107019853.
